# Can ADC changes help mRECIST or LI-RADS treatment response algorithm better diagnose pathological response of HCC after preoperative radiotherapy? Secondary analysis of a prospective phase 2 trial

**DOI:** 10.1186/s13244-025-02090-7

**Published:** 2025-10-16

**Authors:** Rong Cong, Fan Wu, Yirui Zhai, Feng Ye, Shuang Wang, Zhaowei Chen, Bing Feng, Leyao Wang, Yongjian Zhu, Sicong Wang, Xinming Zhao, Bo Chen, Xiaohong Ma

**Affiliations:** 1 Department of Diagnostic Radiology, National Cancer Center/National Clinical Research Center for Cancer/Cancer Hospital, Chinese Academy of Medical Sciences and Peking Union Medical College, Beijing, China; 2 Department of Hepatobiliary Surgery, National Cancer Center/National Clinical Research Center for Cancer/Cancer Hospital, Chinese Academy of Medical Sciences and Peking Union Medical College, Beijing, China; 3 Department of Radiation Oncology, National Cancer Center/National Clinical Research Center for Cancer/Cancer Hospital, Chinese Academy of Medical Sciences and Peking Union Medical College, Beijing, China; 4 General Electric Healthcare (China), Beijing, China

**Keywords:** Hepatocellular carcinoma, Radiotherapy, Response evaluation, Magnetic resonance imaging, Apparent diffusion coefficient

## Abstract

**Objectives:**

To explore the role of apparent diffusion coefficient (ADC) changes in predicting pathological response to preoperative radiotherapy (RT) in hepatocellular carcinoma (HCC) compared to existing evaluation criteria, using histopathology as the reference standard.

**Materials and methods:**

Building on the prospective clinical trial, we included 35 patients with 38 HCCs who underwent preoperative RT followed by hepatectomy between December 2014 and January 2019. Pre- and post-RT ADC parameters (ADC_roi_ and ADC_slice_ measured from representative areas and histogram parameters derived from whole-tumor volume) were compared, and the percentage change of parameters (Δ-parameters%) was calculated to correlate with major pathological response (MPR). The modified Response Evaluation Criteria in Solid Tumors (mRECIST) and Liver Imaging Reporting and Data System Treatment Response (LR-TR) categories were evaluated. ROC analysis was performed to assess discrimination performance.

**Results:**

ADC values, interquartile range, range, variance, mean absolute deviation, robust mean absolute deviation, and root mean squared increased; energy and total energy decreased; and skewness developed into negative skewness after RT. Higher Δ-ADC_roi_%, Δ-ADC_slice_%, Δ-ADC_mean_%, and Δ-ADC_median_% and lower Δ-energy% and Δ-total energy% were associated with MPR. LR-ADC_slice_ showed the best performance, with significantly higher AUC than mRECIST/LR-TR, Δ-ADC_mean_%, and Δ-ADC_median_% (0.917 vs 0.708, 0.732, and 0.705, respectively; *p* = 0.005, 0.029, and 0.023). Responders had significantly better RFS than non-responders according to Δ-ADC_roi_% (*p* = 0.024).

**Conclusions:**

ADC changes have the potential to predict the pathological response of HCC to preoperative RT, thereby enhancing current evaluation criteria. Integration of Δ-ADC_slice_% and LR-TR yielded the best results.

**Critical relevance statement:**

Δ-ADC_slice_%, with high performance in predicting pathological response, excellent inter-observer agreement, and the potential to supplement existing evaluation criteria, is a promising method for determining therapeutic response to preoperative radiotherapy and may facilitate the early indication for further surgery.

**Key Points:**

- Precise assessment of hepatocellular carcinoma response is required for patients undergoing preoperative radiotherapy.
- Radiotherapy induced an increase in ADC values and heightened intratumoral heterogeneity.
- As the delineated region of interest expanded, AUC decreased and inter-observer agreement increased.
- Δ-ADC_slice_% exhibited excellent performance in predicting pathological response and the potential to supplement existing evaluation criteria.

**Graphical Abstract:**

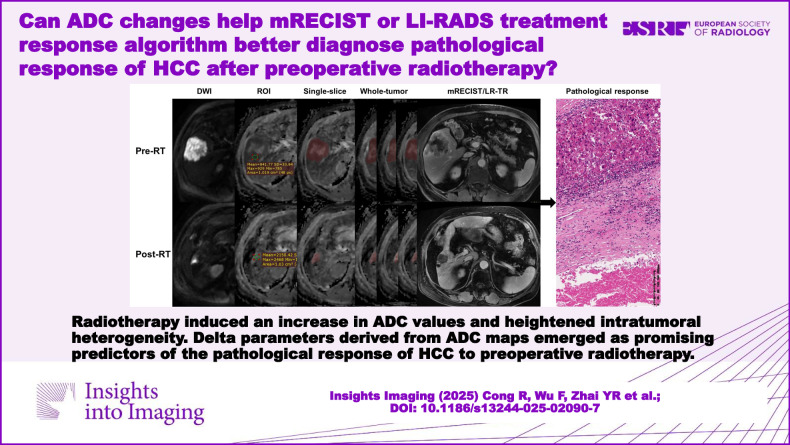

## Introduction

Hepatocellular carcinoma (HCC) poses a significant healthcare challenge globally due to its high morbidity, low resection rate, high mortality, and high recurrence risk [1]. Currently, radical surgical resection stands as the primary approach for ensuring the long-term survival of patients with HCC. However, a substantial number of individuals are initially ineligible for surgery, either due to the advanced disease stage at diagnosis or the high complexity and risk of resection [2, 3].

Locoregional therapy (LRT) plays a crucial role in managing unresectable HCCs or resectable HCCs that are at a high risk of recurrence postoperatively. Effective LRT may eliminate or reduce tumor viability, increase access to surgical intervention, transform incurable cases into curable ones, and ultimately improve patient outcomes [3–5]. Advancements in modern radiotherapy (RT) techniques have significantly expanded the possibilities of using RT as a safe and effective LRT option for HCC [6, 7]. RT has been explored in combination with surgery for HCC, with a previous study indicating the superiority of preoperative RT over postoperative RT in enhancing the long-term survival of patients with HCC [8]. Specifically, patients with portal vein tumor thrombus, centrally located HCC, or a high risk of microvascular invasion stand to benefit from effective preoperative RT [9–11]. As a result, achieving a personalized treatment strategy, heavily reliant on tumor viability post-therapy, necessitates the identification of patients likely to achieve improved tumor control through RT and the accurate evaluation of HCC response to RT.

Noninvasive and preferably early objective evaluation of HCC response to RT is challenging. The modified Response Evaluation Criteria in Solid Tumors (mRECIST) and the Liver Imaging Reporting and Data System treatment response algorithm (LR-TR), both considering arterial phase hyperenhancement (APHE) as indicative of viable tumors, are widely considered to reflect the degree of treatment success and more commonly used in LRT response evaluation for HCC [12]. However, APHE, often seen after stereotactic body radiation therapy (SBRT), does not consistently indicate viable tumors requiring retreatment, potentially limiting the applicability of APHE-based criteria in the early post-RT phase [13, 14]. Diffusion-weighted imaging (DWI) is an attractive option that can be obtained in minutes without needing any contrast agent, and it has shown promise in predicting and monitoring therapeutic response by quantifying the diffusion of water molecules through apparent diffusion coefficient (ADC) [15–17]. Detectable differences in ADC values between viable and necrotic HCC portions post-LRT, as well as before and after LRT, have highlighted its potential utility [18, 19]. Notably, a substantial increase in ADC values was observed as early as day 1 after RT in HCC mouse model [20]. A few studies investigated associations of ADC changes following or during SBRT in HCC with local progression or response [21–24]. However, all of these studies defined response based on RECIST or mRECIST instead of pathological response, and used one or three regions of interest (ROI) instead of the whole-tumor volume for analysis.

Research connecting MRI changes with the pathological response of HCC following RT is currently lacking, and the role and significance of whole-tumor ADC analyses in this context remain unclear. This study aimed to determine the role of delta-parameters derived from pre- and post-RT ADC maps in predicting the pathological response of HCC following RT compared to existing evaluation criteria, using histopathology as the reference standard. Additionally, we aimed to investigate the potential added value of whole-tumor ADC analysis in this predictive framework.

## Materials and methods

### Patients

This study was conducted under a prospective phase 2 nonrandomized controlled trial (ClinicalTrials.gov identifier: NCT02580929) approved by the ethical committee of the Cancer Institute and Hospital of the Chinese Academy of Medical Sciences (Number: NCC2014 YP-22). Written informed consent was obtained from all of the patients before participating in the clinical trial. The clinical trial enrolled a total of 38 patients who underwent RT followed by surgical resection for HCC between December 2014 and January 2019, in accordance with the specific inclusion and exclusion criteria described in the published paper [10]. Among them, patients who underwent MRI within one month before RT and another MRI within two months before surgery were further included in this study. After excluding two patients lacking available MRI data before RT and one with infiltrative lesions, 35 patients with 38 lesions were ultimately enrolled.

### Image acquisition

All MR examinations were conducted before and after RT using 3.0-T scanners (Signa HDx or Discovery MR 750; GE Medical System, Milwaukee, WI, USA). The routine liver MRI protocol included in- and opposed-phase T1-weighted imaging (T1WI), axial fat-suppression T2-weighted imaging (T2WI), diffusion-weighted imaging, and dynamic contrast-enhanced T1-weighted three-dimensional spoiled gradient-echo liver acceleration volume acquisition. DWI was performed using two b-values (0 and 800 s/mm^2^), demonstrating acceptable image quality and signal-to-noise ratio to ensure reliable ADC measurements. ADC maps were automatically generated from DWI using Functool software (ADW4.6, GE Healthcare). Detailed information regarding image acquisition is provided in Supplementary A1 and Table S1.

### Therapeutic procedures

All patients underwent preoperative intensity-modulated radiotherapy (IMRT). The prescription dose to 95% of the planning target volume ranged from 50 to 60 Gy, administered in 25–30 fractions for 5–6 weeks according to the dose constraints of organs at risk. Surgery procedures were carried out following extensive discussion within a multidisciplinary team. Detailed information regarding RT and surgical procedures has been described in the published study [10].

### Image analysis

ADC measurements and radiological response evaluations were independently conducted by two radiologists (i.e., readers 1 and 2, with 3 and 8 years of experience in abdominal imaging, respectively) who were blinded to the pathologic tumor viability throughout the assessment process.

#### Quantitative image analysis

After discussing and reaching a consensus regarding ADC quantification, the readers measured ADC values of tumors on both pre- and post-RT ADC maps utilizing three distinct methods. To ensure accuracy, pre- and post-RT MRI were compared to confirm that the ROIs were situated within the primary tumor.

For the circular ROI method, the ROIs were positioned within the most cellular tumor areas (i.e., areas with the most prominent restrictive diffusion on both DWI and the corresponding ADC map, excluding T2 shine-through and blackout areas) and ROIs placement was determined by lesion size as following: place one ROI in the largest slice for lesions < 2 cm; two ROIs in the two largest consecutive slices for lesions 2–4 cm; three ROIs in the three largest consecutive slices for lesions > 4 cm [25]. Mean ADC was used for lesions with more than one ROI. Subsequent analyses utilized the average values provided by the two readers, collectively recorded as ADC_roi_.

For single-slice ADC measurements, radiologists determined ADC values by outlining the largest cross-sectional area of the lesion, including cystic, necrotic, and hemorrhagic regions. The average value determined by the two readers was recorded as the ADC_slice_ for further analysis.

For whole-tumor ADC histogram analysis, ADC maps were imported into ITK-SNAP software (version v.3.8.0, www.itksnap.org) for manual three-dimensional segmentation. Reader 1 drew ROIs along the tumor border on all slices of the ADC map, utilizing T2WI, DWI, and DCE-T1WI as references. The ROIs encompassed the entire tumor area, including cystic, necrotic, and bleeding regions, to capture tumor heterogeneity comprehensively. Reader 2 independently performed tumor segmentations to assess interobserver agreement for ADC histogram metrics. The histogram metrics obtained by Reader 1 were used for subsequent analysis.

The first-order histogram parameters included ADC_mean_, ADC_median_, 10th percentile ADC (ADC_10th_), 90th percentile ADC (ADC_90th_), ADC_min_, ADC_max_, interquartile range (IQR), range, variance, mean absolute deviation (MAD), robust mean absolute deviation (rMAD), root mean square (RMS), energy, total energy, entropy, skewness, kurtosis, and uniformity.

To quantify the percentage change of each parameter (Δ-parameter%), the following formula was applied for each lesion:

$$\Delta {\mbox{-}}{{\rm{parameter}}} \% =\frac{{{{\rm{parameter}}}}_{{{\rm{post}}}{\mbox{-}}{{\rm{RT}}}}-{{{\rm{parameter}}}}_{{{\rm{pre}}}{\mbox{-}}{{\rm{RT}}}}}{{{{\rm{parameter}}}}_{{{\rm{pre}}}{\mbox{-}}{{\rm{RT}}}}}\times 100$$

#### Radiological response evaluation

Two radiologists who were blinded to the pathologic tumor viability independently reviewed the pre- and post-RT MRI. They measured the single longest diameter (SLD) of the enhancing portion of the lesion during the arterial phase (indicative of viable tumor tissue), carefully avoiding necrotic areas, following the mRECIST guidelines [26]. The mRECIST categories, including complete response (CR), stable disease (SD), partial response (PR), and progressive disease (PD), were assigned based on the percentage change in the SLD of viable tumor tissue. On post-RT MRI, the radiologists assessed the viability of each lesion using the LR-TR. LR-TR categories included (1) no lesional enhancement or expected treatment-specific enhancement pattern, categorized as LR-TR nonviable; (2) nodular, masslike, or thick irregular tissue in or along the treated lesion with any of the following findings: APHE, washout, or enhancement similar to pretreatment, categorized as LR-TR viable; and (3) indeterminate enhancement, categorized as LR-TR equivocal. Interobserver agreement was also assessed. Any discrepancies between the two radiologists were resolved through joint review until a consensus was reached.

### Pathology analysis

The pathological assessments were conducted by a pathologist with 10 years of experience in gastrointestinal pathology. The pathological response was evaluated by determining the percentage of the surface area occupied by nonviable tumor cells (represented by necrosis or fibrosis) relative to the total tumor area. A major pathological response (MPR) was defined as the presence of 10% or fewer viable tumor cells in the primary tumor. MPR encompassed a pathologic complete response (pCR), which was characterized by the absence of residual viable tumor cells in the resected liver specimen.

### Follow-up

Recurrence-free survival (RFS) was computed from the date of surgery to the earliest identification of recurrence or the last follow-up. The final follow-up for this study was scheduled for October 31, 2022. Recurrence events included both intrahepatic tumor relapse and extrahepatic metastasis.

### Statistical analysis

Interobserver agreement was evaluated using two-way mixed absolute agreement intraclass correlation coefficients (ICCs) for ADC parameters, and weighted κ statistics were employed for the mRECIST and LR-TR categories. The categorization of the agreement levels was as follows: poor agreement (0–0.20), fair agreement (0.21–0.40), moderate agreement (0.41–0.60), good agreement (0.61–0.80), and excellent agreement (0.81–1.00). After assessing normality with the Shapiro–Wilk test, continuous variables were compared using the paired sample *t*-test or paired Wilcoxon test between pre- and post-RT MRI, and Student’s *t*-test or Mann–Whitney U test between the MPR and non-MPR groups. Receiver operating characteristic (ROC) curves analysis was performed for significant Δ-parameters% to evaluate discrimination performance and determine the optimal cutoff value based on the Youden index. Survival curves were generated using the Kaplan–Meier method and compared using the log-rank test. All statistical analyses were performed using SPSS software (version 25.0, IBM) and MedCalc software (version 20.0.4). A two-sided *p* < 0.05 was considered statistically significant.

## Results

### Clinical characteristics and histopathologic findings

A total of 35 patients (mean age: 55.0 ± 9.8 years; 32 males) with 38 lesions (median size on imaging = 5.0 cm, interquartile range = 4.0–7.8 cm) were evaluated in this study. The clinical characteristics of the enrolled patients are summarized in Table 1. Histopathological analysis of surgical specimens showed MPR in 12 lesions, including pCR in six lesions. No difference in clinical characteristics was observed between the MPR and non-MPR groups (Table 1). The median time interval between the pre-RT MRI and RT initiation was 0.9 months (range: 0.4–1.0 months). The period between post-RT MRI (the last examination before surgery) and surgery was 0.6 months (range: 0.1–1.5 months).

**Table 1:** Patients’ clinical characteristics

Characteristics	All patients (*n* = 35)	MPR group* (*n* = 10)	Non-MPR group (*n* = 25)	*p*-value
Age, years	58 (47–62)	49 (46–65.25)	58 (47–62)	0.680
Sex, male	32 (91.4)	9 (90.0)	23 (92.0)	1.000
Hepatitis B	28 (80.0)	8 (80.0)	20 (80.0)	1.000
Hepatitis C	1 (2.9)	1 (10.0)	0 (0.0)	0.286
Child-Pugh class A	35 (100.0)	10 (100.0)	25 (100.0)	-
Liver cirrhosis	26 (74.3)	9 (90.0)	17 (68.0)	0.235
BCLC stage				
A	29 (82.9)	7 (70.0)	22 (88.0)	0.322
B	3 (8.6)	1 (10.0)	2 (8.0)	1.000
C	3 (8.6)	2 (20.0)	1 (4.0)	0.190
Pretreatment TBIL (μmol/L)	11.7 (9.6–17.1)	11.2 (9.4–14.4)	11.9 (9.8–17.4)	0.636
Pretreatment albumin (g/L)	45.5 (42.7–47.4)	44.6 (42.6–46.1)	45.7 (42.5–48.0)	0.355
Pretreatment AFP (ng/mL)	106.0 (11.3–2537.0)	265.8 (7.6–8226.5)	93.3 (15.0–1423.0)	0.770
Preoperative AFP (ng/mL)	14.6 (5.5–121.5)	11.3 (3.8–74.0)	15.8 (6.6–148.4)	0.228
Number of lesions				1.000
1	32 (91.4)	9 (90.0)	23 (92.0)	
2	3 (8.6)	1 (10.0)	2 (8.0)	
Size of the largest tumor, cm				0.709
< 5	13 (37.1)	3 (30.0)	10 (40.0)	
≥ 5	22 (62.9)	7 (70.0)	15 (60.0)	

Continuous variables are presented as median, with interquartile range in parentheses, and categorical variables are the number of patients, with percentages in parentheses

*BCLC* Barcelona Clinical Liver Cancer classification, *TBIL* total bilirubin, *AFP* alpha-fetoprotein, *MPR* major pathological response

* Patients with all lesions in major pathological response

### Comparison of ADCmap-derived parameters between pre- and post-RT

The ICCs demonstrated good agreement for the pre-RT ADC_roi_(0.774 [95% CI: 0.611–0.874]) and excellent agreement for the post-RT ADC_roi_, pre-RT ADC_slice_, and post-RT ADC_slice_(0.850 [95% CI: 0.734–0.917], 0.890 [95% CI: 0.798–0.941], and 0.865 [95% CI: 0.756–0.927], respectively). The ICCs for pre- and post-RT histogram metrics ranged from good to excellent, with values between 0.772 and 0.997 and 0.721 and 0.973, respectively (Table S2).

**Fig. 1:**
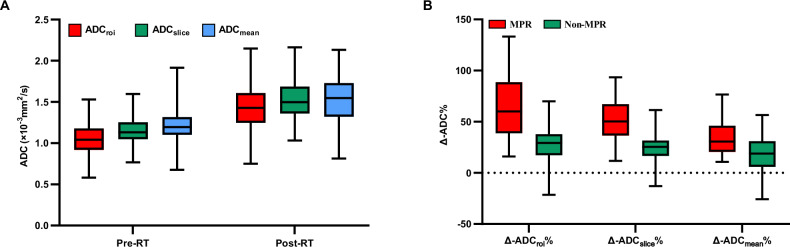
Box and whisker plots represent the distribution of pre- and post-RT ADC values (**A**) and the percentage change of ADC values obtained with three ROI drawing methods in major pathological response (MPR) and non-MPR groups (**B**)

**Fig. 2:**
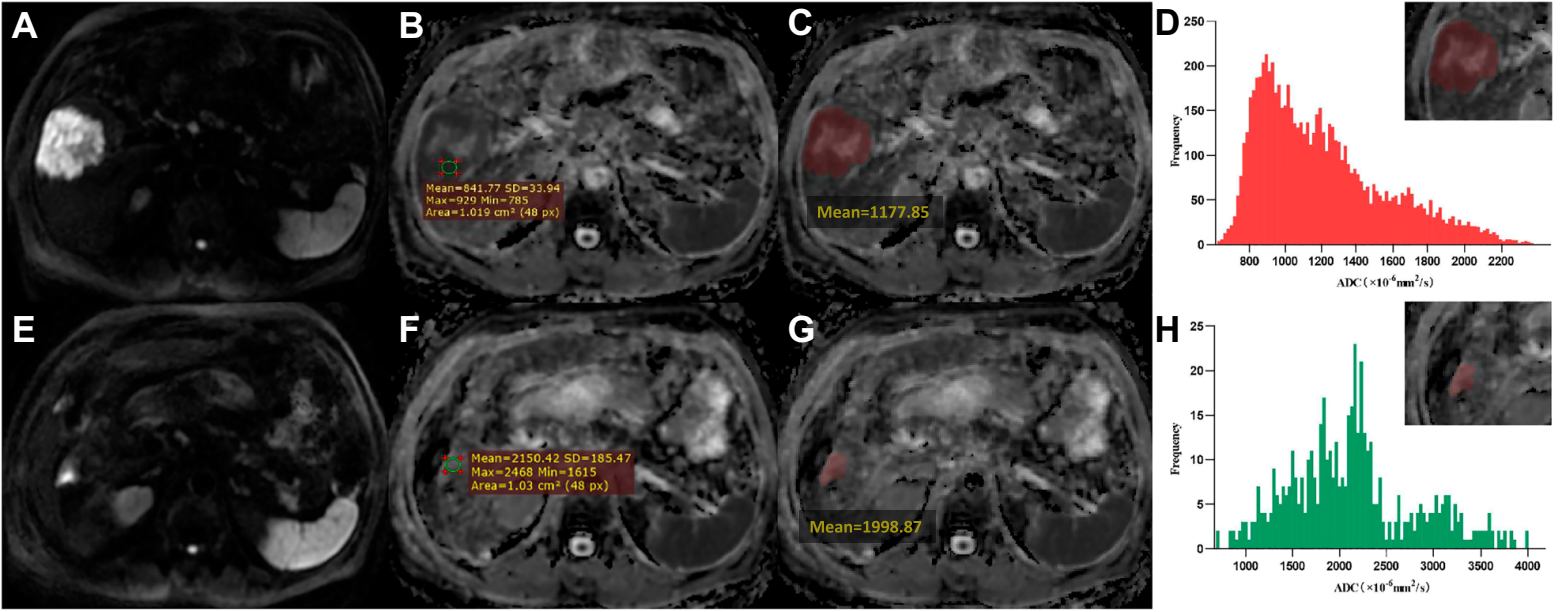
A 74-year-old male achieved pathologic complete response (pCR) to preoperative radiotherapy. **A**, **E** DWI images (b = 800 s/mm^2^) before and after RT. **B**, **F** ADC maps for circular ROI ADC measurements before and after RT show a 155% increase in ADC_roi_. **C**, **G** ADC maps for single-slice ADC measurements before and after RT show a 70% increase in ADC_slice_. **D**, **H** The whole-tumor ADC histogram analysis before and after RT shows an increase in ADC values and their dispersion and a decrease in skewness

Significant increments were observed in ADC values, including ADC_roi_, ADC_slice_, ADC_mean_, ADC_median,_ ADC_10th_, ADC_90th_, ADC_min_, and ADC_max_, after RT (all *p* < 0.05). Similarly, the IQR, range, variance, MAD, rMAD, and RMS were significantly higher after RT than before RT (all *p* < 0.05). Additionally, there was a decrease in energy and total energy, and a negative skewness shift in skewness. However, no significant changes were noted in entropy, kurtosis, or uniformity after treatment (all *p* > 0.05) (Table 2, Figs. 1A, 2).

**Table 2:** Comparison of pre-and post-RT ADC map-derived parameters

Parameters	Pre-RT MRI	Post-RT MRI	*p*-value
ADC_roi_ (×10^−^^3^ mm^2^/s)	1.07 ± 0.21	1.45 ± 0.28	< 0.001*
ADC_slice_ (×10^−^^3^ mm^2^/s)	1.17 ± 0.19	1.53 ± 0.25	0.009*
ADC_mean_ (×10^−^^3^ mm^2^/s)	1.20 (1.10–1.32)	1.51 ± 0.27	< 0.001*
ADC_median_ (×10^−^^3^ mm^2^/s)	1.17 (1.08–1.32)	1.50 ± 0.27	< 0.001*
ADC_10th_ (×10^−^^3^ mm^2^/s)	0.93 ± 0.23	1.13 ± 0.28	< 0.001*
ADC_90th_ (×10^−^^3^ mm^2^/s)	1.48 (1.38–1.67)	1.88 (1.71–2.07)	< 0.001*
ADC_min_ (×10^−^^3^ mm^2^/s)	0.50 ± 0.32	0.66 ± 0.42	0.007*
ADC_max_ (×10^−^^3^ mm^2^/s)	2.20 (1.95–2.60)	2.42 (2.05–2.93)	0.036*
IQR (×10^−^^3^ mm^2^/s)	0.31 (0.24–0.38)	0.40 ± 0.15	0.003*
Range (×10^−^^3^ mm^2^/s)	0.68 (0.38–0.96)	1.82 (1.10–2.82)	< 0.001*
Variance	5.52 × 10^4^ (3.86 × 10^4^–8.69 × 10^4^)	8.88 × 10^4^ (4.21 × 10^4^–1.61 × 10^5^)	0.008*
MAD	184.38 (157.14–231.66)	245.51 ± 93.24	0.004*
rMAD	130.39 (103.85–154.52)	169.58 ± 65.84	0.003*
RMS	1490.48 (1368.01–1622.54)	1777.49 ± 326.03	< 0.001*
Energy	7.84 × 10^9^ (2.05 × 10^9^–1.76 × 10^10^)	3.61 × 10^9^ (5.91 × 10^8^–1.42 × 10^10^)	0.010*
Total energy	1.11 × 10^11^ (3.08 × 10^10^–2.67 × 10^11^)	6.86 × 10^10^ (9.66 × 10^9^–2.34 × 10^11^)	0.007*
Skewness	0.48 ± 0.50	0.04 (−0.27 to 0.44)	0.001*
Entropy	7.45 ± 0.56	7.44 (6.66–8.05)	0.690
Kurtosis	3.72 (2.96–4.25)	3.28 (2.54–4.32)	0.388
Uniformity	0.0076 (0.0054–0.0092)	0.0071 (0.0046–0.0112)	0.690

Data were expressed as mean ± standard deviation (normal distribution) or median with interquartile range (non-normal distribution)

*ADC* apparent diffusion coefficient, *RT* radiotherapy, *MRI* magnetic resonance imaging, *IQR* interquartile range, *MAD* mean absolute deviation, *rMAD* robust mean absolute deviation, *RMS* root mean square

* *p* < 0.05

### Associations between Δ-parameters% and MPR

The percentage changes in ADC_roi_, ADC_slice_ and histogram metrics from the whole-tumor volume between pre- and post-RT measurements in the MPR and non-MPR groups are presented in Table 3. Δ-ADC_roi_%, Δ-ADC_slice_%, Δ-ADC_mean_%, and Δ-ADC_median_% were significantly higher in the MPR group compared to the non-MPR group (64.3% vs 27.6%, 50.9% vs 24.9%, 34.9% vs 17.8%, and 36.0% vs 19.5%, *p* < 0.05) (Figs. 1B, 2). Δ-ADC_10th_%, Δ-ADC_90th_%, Δ-ADC_min_%, Δ-ADC_max_%, Δ-range%, Δ-variance%, Δ-MAD%, Δ-rMAD%, and Δ-RMS% derived from whole-tumor volume tended to be higher in the MPR group but did not show significant differences between the two groups (all *p* > 0.05). Patients in the MPR group also exhibited a higher decrease in Δ-energy% (*p* = 0.006) and Δ-total energy% (*p* = 0.004) compared to those in the non-MPR group.

**Table 3:** Comparison of Δ-parameters% between the MPR and the non-MPR groups

Δ-parameters%	MPR group	Non-MPR group	*p*-value
Δ-ADC_roi_%	64.3 ± 34.1	27.6 ± 20.3	< 0.001*
Δ-ADC_slice_%	50.9 ± 23.5	24.9 ± 15.7	< 0.001*
Δ-ADC_mean_%	34.9 ± 18.9	17.8 ± 20.7	0.020*
Δ-ADC_median_%	36.0 ± 22.8	19.5 ± 21.3	0.037*
Δ-ADC_10th_%	28.3 ± 35.6	21.4 ± 28.5	0.525
Δ-ADC_90th_%	30.8 ± 21.0	19.2 ± 22.1	0.136
Δ-ADC_min_%	50.0 (10.0–129.7)	27.0 (−21.9 to 96.8)	0.233
Δ-ADC_max_%	21.4 ± 29.1	9.1 ± 24.2	0.177
Δ-IQR%	48.8 ± 50.8	29.7 ± 62.0	0.356
Δ-range%	7.6 ± 48.4	4.8 ± 40.5	0.853
Δ-variance%	145.8 (1.0–235.1)	29.8 (−28.1 to 152.1)	0.187
Δ-MAD%	45.0 ± 48.9	23.6 ± 42.3	0.221
Δ-rMAD%	48.8 ± 53.6	28.2 ± 58.4	0.307
Δ-RMS%	26.2 ± 18.7	16.3 ± 17.5	0.118
Δ-energy%	−70.5 (−87.6 to −49.9)	−17.1 (−52.3 to 19.7)	0.006*
Δ-total energy%	−71.6 (−87.3 to −53.1)	−8.3 (−44.8–20.6)	0.004*
Δ-skewness%	−114.3 (−260.4 to −48.0)	−94.2 (−171.6 to −15.1)	0.470
Δ-entropy%	0.4 (−24.8 to 4.6)	0.0 (−5.0 to 7.5)	0.245
Δ-kurtosis%	−3.8 ± 52.9	0.7 ± 36.1	0.760
Δ-uniformity%	0.4 (−21.7 to 192.6)	−0.8 (−29.2 to 28.7)	0.233

Data were expressed as mean ± standard deviation or median with interquartile range

*ADC* apparent diffusion coefficient, *MPR* major pathological response, *IQR* interquartile range, *MAD* mean absolute deviation, *rMAD* robust mean absolute deviation, *RMS* root mean square

* *p* < 0.05

### Diagnostic performance for identifying MPR

**Fig. 3:**
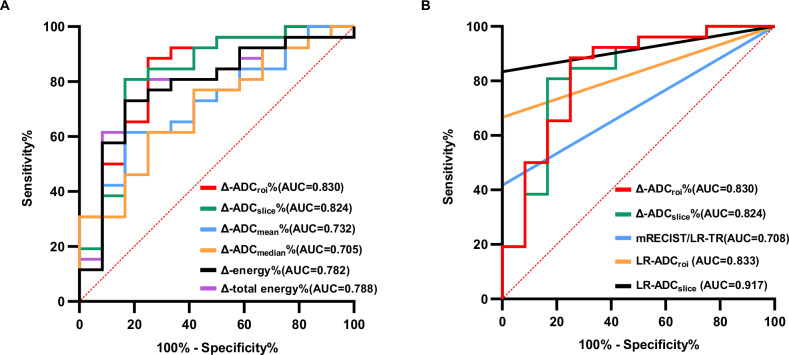
Receiver operating characteristics (ROC) curve analysis of Δ-parameters% (**A**) and evaluation criteria (**B**) for identifying MPR

Δ-ADC_roi_%, Δ-ADC_slice_%, Δ-ADC_mean_%, Δ-ADC_median_%, Δ-energy%, and Δ-total energy% all exhibited relatively high area under the curve (AUC) values in discriminating MPR from non-MPR (respective AUCs were 0.830 [95% CI: 0.673–0.932], 0.824 [95% CI: 0.666–0.928], 0.732 [95% CI: 0.564–0.863]), 0.705 [95% CI: 0.535–0.842], 0.782 [95% CI: 0.619–0.899] and 0.788 [95% CI: 0.626–0.904] (Table 4 and Fig. 3A). Δ-ADC_roi_% and Δ-ADC_slice_% outperformed the percentage changes of histogram parameters from whole-tumor volume, although no statistically significant differences were observed.

**Table 4:** Performance of Δ-parameters% and evaluation criteria for identifying MPR

Methods	Sensitivity (%)	Specificity (%)	PPV (%)	NPV (%)	Accuracy (%)	AUC (95% CI)	Cutoff value	*p*-value
Δ-ADC_roi_%	75 (9/12)	88 (23/26)	75 (9/12)	88 (23/26)	84 (32/38)	0.830 (0.673, 0.932)	+46	< 0.001*
Δ-ADC_slice_%	83 (10/12)	81 (21/26)	67 (10/15)	91 (21/23)	82 (31/38)	0.824 (0.666, 0.928)	+33	< 0.001*
Δ-ADC_mean_%	83 (10/12)	62 (16/26)	50 (10/20)	89 (16/18)	68 (26/38)	0.732 (0.564, 0.863)	+19	0.005*
Δ-ADC_median_%	75 (9/12)	62 (16/26)	47 (9/19)	84 (16/19)	66 (25/38)	0.705 (0.535, 0.842)	+21	0.023*
Δ-energy%	83 (10/12)	73 (19/26)	59 (10/17)	90 (19/21)	76 (29/38)	0.782 (0.619, 0.899)	−48	0.001*
Δ-total energy%	83 (10/12)	77 (20/26)	63 (10/16)	91 (20/22)	79 (30/38)	0.788 (0.626, 0.904)	−53	< 0.001*
mRECIST CR/ LR-TR nonviable	42 (5/12)	100 (26/26)	100 (5/5)	79 (26/33)	82 (31/38)	0.708 (0.539, 0.844)		
LR-ADC_roi_ CR	67 (8/12)	100 (26/26)	100 (8/8)	87 (26/30)	89 (34/38)	0.833 (0.677, 0.934)		
LR-ADC_slice_ CR	83 (10/12)	100 (26/26)	100 (10/10)	93 (26/28)	95 (36/38)	0.917 (0.780, 0.981)		

*MPR* major pathological response, *PPV* positive predictive value, *NPV* negative predictive value, *AUC* area under the curve, *ADC* apparent diffusion coefficient, *IQR* interquartile range, *MAD* mean absolute deviation, *mRECIST* modified Response Evaluation Criteria In Solid Tumors, *CR* complete response, *LR-TR* Liver Imaging Reporting and Data System treatment response algorithm

* *p* < 0.05

At the last MR examination before surgery, 5/38 (13.2%), 14/38 (36.8%), and 19/38 (50%) lesions were categorized as CR, PR, and SD, respectively, according to the mRECIST, and 5/38 (13.2%), 6/38 (15.8%), and 27/38 (71.1%) were categorized as nonviable, equivocal, and viable according to LR-TR respectively. Interobserver agreement for mRECIST and LR-TR was good (κ = 0.733 [95% CI: 0.580–0.885], κ = 0.781 [95% CI: 0.640–0.992], respectively). mRECIST CR and LR-TR nonviable showed equal AUC (0.708 [95% CI: 0.539–0.844]), lower but not significantly different from the AUC of Δ-ADC_roi_% (*p* = 0.145) or Δ-ADC_slice_% (*p* = 0.270). LR-TR nonviable or LR-TR equivocal with Δ-ADC_roi_% > 46% was defined as LR-ADC_roi_ CR, and LR-TR nonviable or LR-TR equivocal with Δ-ADC_slice_% > 33% was defined as LR-ADC_slice_ CR. LR-ADC_slice_ CR demonstrated the best performance with an AUC of 0.917 (95% CI: 0.780–0.981), significantly higher than mRECIST/LR-TR (*p* = 0.005), Δ-ADC_mean_% (*p* = 0.029), and Δ-ADC_median_% (*p* = 0.023) (Table 4 and Fig. 3B). LR-ADC_roi_ CR exhibited no significant differences compared to mRECIST/LR-TR and Δ-parameters%. Δ-parameters% showed a tendency of higher sensitivity and lower specificity at optimal cutoff values compared to mRECIST and LR-TR.

### Postoperative outcomes

**Fig. 4:**
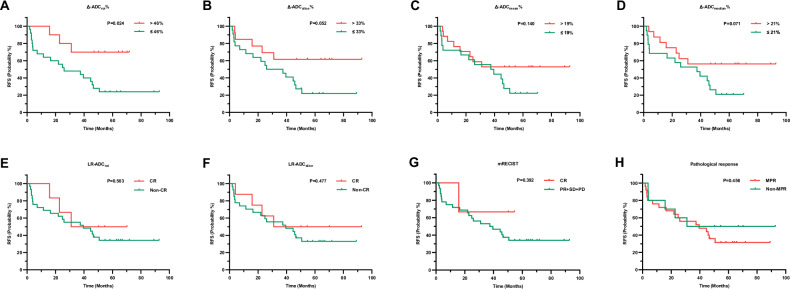
Kaplan–Meier curves for RFS of 35 patients grouped by Δ-ADC_roi_% (**A**), Δ-ADC_slice_% (**B**), Δ-ADC_mean_% (**C**), Δ-ADC_median_% (**D**), LR-ADC_roi_ (**E**), LR-ADC_slice_ (**F**), mRECIST (**G**), and pathological response (**H**)

The median RFS was 39.5 months (IQR: 11.5–58.3 months), with 1-year, 3-year, and 5-year RFS rates of 74.3%, 54.3%, and 36.7%, respectively. A further comparison of RFS between responders and non-responders according to different criteria and the above-mentioned Δ-parameters% based on the cutoff value was made. Assigning the worst lesion response to each patient reveals the patient’s response. Patients with a > 46% increase in ADC_roi_ showed significantly longer RFS compared to those without (*p* = 0.024 by log-rank test), while those with a > 33% increase in ADC_slice_ also demonstrated borderline significantly prolonged RFS (*p* = 0.052 by log-rank test) (Fig. 4A, B). Patients with > 19% ADC_mean_ increase, > 21% ADC_median_ increase, LR-ADC CR, mRECIST CR, or MPR showed non-significant RFS prolongation trends (all *p* > 0.05 by log-rank test) (Fig. 4C–H). Responders grouped by Δ-energy% and Δ-total energy% showed a trend toward worse RFS than non-responders (*p* = 0.099 and 0.223 by log-rank test) (Fig. S1).

## Discussion

Building on the initial phase II prospective clinical trial investigating preoperative IMRT combined with liver radical resection for centrally located HCC [10], we further analyzed whether percentage changes in ADC parameters before and after IMRT could predict the pathological response of HCC. Additionally, we explored the utility of three quantitative measurement methods. Our findings revealed that higher increases in ADC_roi_, ADC_slice_, ADC_mean_, and ADC_median_, as well as higher decreases in energy and total energy, were related to MPR. The percentage changes in these associated variables demonstrated relatively high AUC values for predicting MPR, surpassing the performance of mRECIST and reinforcing the LR-TR.

Effective preoperative RT has been identified as a strategy to diminish portal vein tumor thrombus, minimize the risk of positive surgical margins, and reduce early postoperative recurrence [9–11]. In the clinical cohort, patients undergoing preoperative IMRT and surgery achieved a 5-year overall survival rate of 69.1% and a partial response rate of 42.1% [10]. Nevertheless, clinicians require an early and precise assessment of tumor control to guide further therapeutic decisions after RT. DWI-derived ADC was highlighted for quantifying the diffusion of water molecules, offering microstructural insights into cell density and cell membrane integrity. The intra-tumor changes induced by treatment were reflected in alterations in DWI signal intensity and ADC value. Previous research suggested that DWI with ADC maps could enhance the detection of viable HCC tumors treated with RT [27], and a significant increase in ADC values was observed early after RT in HCC [20, 28]. The ADC histogram metrics from the whole-tumor volume was noted to characterize intratumoral heterogeneity, which also exhibited dynamic changes during treatment [29]. Consequently, ADC holds promise for noninvasive and early objective evaluation of treatment response without needing any contrast agent. The additional value of ADC-based quantitative analysis compared to existing APHE-based criteria for evaluating the RT response of HCC remains uncertain, and using pathological response as a reference is required.

In this study, the ADC values increased significantly after RT, with Δ-ADC_roi_% and Δ-ADC_slice_% demonstrating comparably high diagnostic performance in identifying MPR. The observed rise in ADC values may be attributed to processes such as tumor cell death, reduced proliferation, and the influx of immune cells after irradiation [20]. These processes lead to the loss of cellular integrity and reduced tumor density, allowing for greater water molecule diffusion. The percentage change in mean ADC values obtained through three distinct measurement methods all exhibited a correlation with MPR. Notably, as the delineated region of interest expanded, the AUC decreased. Δ-ADC_mean_% derived from whole-tumor volume did not present additional advantages over Δ-ADC_roi_% and Δ-ADC_slice_%. A study by Blazic et al [30] analyzing the impact of different ADC measurement methods on assessing pCR to neoadjuvant CRT in patients with rectal cancer found that the single-section and whole-tumor volume methods were equally accurate in discriminating pCR from non-pCR, outperforming the three ROI methods. The superior performance of the whole-tumor volume method observed in their study was inconsistent with our results, possibly due to the higher and more complex heterogeneity of HCC compared to rectal cancer. The inclusion of pre-RT intratumoral necrosis and bleeding in the ROI might have influenced the calculation of percentage changes in mean ADC values, potentially diminishing the impact of changes in viable tumor cells. This suggests that, for HCC, changes in ADC after RT within regions of the tumor cells, even in a small representative area within the lesion, may offer better predictive value for major pathological response. Furthermore, from the perspective of interobserver agreement, the single-slice method demonstrated superior repeatability compared to the circular ROI method and comparable repeatability to the whole-tumor volume method. These findings suggest that the relatively fast single-section method might be the most practical approach in clinical settings, which is in line with the perspective of Blazic et al [30].

Other histogram metrics from the whole-tumor volume did not demonstrate an absolute necessity compared to conventional ADC measurements in this study. Energy and total energy exhibited a significant decrease after RT, and their percentage changes outperformed Δ-ADC_mean_% and Δ-ADC_median_% from the whole volume, even though they were still inferior to Δ-ADC_roi_% and Δ-ADC_slice_%. The dependence of energy and total energy on voxel values, numbers, and volumes might probably explain their performance [31]. A significant correlation was observed between Δ-total energy% and the percentage change in tumor diameter (correlation coefficient = 0.844), suggesting that the substantial tumor shrinkage in the MPR group could contribute to its favorable performance in this study. However, its utility in predicting response without significant tumor size changes remains unclear. Metrics such as IQR, range, variance, MAD, rMAD, and RMS, measuring the amount of dispersion, exhibited a significant increase after RT. This increase in the dispersion may be attributed to the diverse and heterogeneous histology of treated tumors, including viable tumor cells, necrosis, and tumor stroma, such as fibrosis and inflammation. However, the inherent high heterogeneity of HCC might render the correlation between these changes and treatment responses less significant.

Comparing the predictive ability of Δ-parameter% and enhancement criteria for pathological viability, Δ-parameters% exhibited higher sensitivity compared to mRECIST and LR-TR. Only five out of the 12 lesions with MPR were categorized as mRECIST CR and LR-TR nonviable. Another five lesions with MPR were categorized as LR-TR equivocal due to various imaging features, such as APHE and post-arterial phase hyperenhancement, or heterogeneous hyperintensity on T1WI. The complexity of signal manifestations after treatment, coupled with the absence of subtracted T1WI images, often leads to observer uncertainty in determining APHE and a lower confidence in declaring nonviability. By incorporating Δ-ADC_roi_% or Δ-ADC_slice_% into the LR-TR equivocal category, the performance of LR-TR significantly improved, especially when fused with Δ-ADC_slice_%. ADC, considered an objective and quantitative indicator, undergoes early changes after treatment, potentially increasing the certainty of nonviability and sensitively detecting responders. Additionally, DWI is a rapid imaging modality suitable for patients in whom contrast agents cannot be used. Further, we identified that patients classified as responders based on Δ-ADC_roi_% and Δ-ADC_slice_% showed longer RFS, outperforming other Δ-parameters%, evaluation criteria, and even pathological response assessments. Notably, five patients achieved MPR; however, all experienced recurrence within 3 years after surgery, which may be due to high preoperative or rising AFP levels. Previous studies have also suggested the prognostic value of ADC changes, with Yu et al [21] reporting a 20% increment of ADC after RT being linked to local progression-free survival and Lo et al [24] finding an ADC change ≥ 25% as a predictor of favorable in-field control, consistent with our findings. Different ROI placements and endpoints also lead to variations among studies in ADC percentage changes post-RT. Nevertheless, it is worth mentioning that long-term follow-up studies are required to validate these findings and identify prognostic factors.

This study has some limitations. First, the single-center design and the limited sample size were notable constraints. Nevertheless, the patients included in our study received more uniform treatment without the implementation of other forms of LRT before and after RT. Second, the small number of patients achieving pCR necessitated grouping patients according to MPR instead of pCR. The limited proportion of patients achieving MPR or pCR may be attributed to the relatively short interval between RT and surgery. Third, the cutoff values utilized in this study were not pre-specified and were determined based on data from this test set, potentially leading to an overestimation of the diagnostic performance of parameters. Therefore, further large-scale, multi-center research is needed to validate and extend our findings. Finally, the monoexponential model was employed to derive ADC in this study. The influence of pseudo-diffusion caused by perfusion on ADC values and the findings is uncertain, warranting future investigation into the added value of intravoxel incoherent motion imaging.

## Conclusions

From a whole-tumor perspective, we observed an increase in ADC values and heightened intratumoral heterogeneity induced by RT. Delta parameters derived from ADC maps emerged as promising predictors of the pathological response of HCC to preoperative RT. Histogram metrics from the whole-tumor volume did not demonstrate an absolute necessity. The percentage change of ADC_roi_ and ADC_slice_ demonstrated excellent performance in estimating MPR with heightened sensitivity versus mRECIST/LR-TR, and the highest accuracy was achieved by incorporating Δ-ADC_slice_% into LR-TR, facilitating the early indication for further surgery. Responses measured by Δ-ADC_roi_% and Δ-ADC_slice_% were also correlated with improved RFS, suggesting their potential as prognostic indicators.

## Supplementary information

ELECTRONIC SUPPLEMENTARY MATERIAL

## Data Availability

The data generated or analyzed during the current study are not publicly available due to the ethics policies of the hospital, but are available from the corresponding author on reasonable request.

## Abbreviations

ADC: Apparent diffusion coefficient
APHE: Arterial phase hyperenhancement
AUC: Area under the curve
CR: Complete response
DWI: Diffusion-weighted imaging
HCC: Hepatocellular carcinoma
IMRT: Intensity-modulated radiotherapy
IQR: Interquartile range
LRT: Locoregional therapy
LR-TR: Liver Imaging Reporting and Data System Treatment Response
MAD: Mean absolute deviation
MPR: Major pathological response
mRECIST: Modified response evaluation criteria in solid tumors
PR: Partial response
RFS: Recurrence-free survival
rMAD: Robust mean absolute deviation
RMS: Root mean square
ROC: Receiver operating characteristic
ROI: Region of interest
RT: Radiotherapy
SBRT: Stereotactic body radiation therapy
SD: Stable disease
