# Supplementary material for: Can ADC changes help mRECIST or LI-RADS treatment response algorithm better diagnose pathological response of HCC after preoperative radiotherapy? Secondary analysis of a prospective phase 2 trial
Source: Insights Imaging. 2025 Oct 16;16:220. doi: 10.1186/s13244-025-02090-7 (PMC12532537; doi:10.1186/s13244-025-02090-7)
Supplement: Supplementary file 1 — ELECTRONIC SUPPLEMENTARY MATERIAL [file 13244_2025_2090_MOESM1_ESM.docx]

Supplemental Material

Materials and Methods

Supplementary A1 Image acquisition

For dynamic contrast-enhanced T1-weighted imaging, unenhanced, arterial phases (30s), portal venous phase (60s), delayed phase/transitional phase (180s), and hepatobiliary phase (20min, only for gadoxetic acid) were acquired before and after injection of gadodiamide (Omniscan 0.5 mmol/ml; GE Healthcare) at a dose of 0.2 mL/kg with a rate of 2 mL/s or gadoxetic acid (Primovist, Bayer Healthcare) at a dose of 0.1 mL/kg with a rate of 1 mL/s followed by a 20 mL 0.9% saline flush via an auto-injector.

The signal-to-noise ratio (SNR) of diffusion-weighted image (DWI) at b value of 800 s/mm2 was calculated using the following formula: SNR = SI_HCC or liver_/SD_liver_, where SI_HCC_ is the signal intensity (SI) of HCC by drawing a circular region of interest (ROI) within the homogeneous area of the largest tumor, SI_liver_ is the SI of liver parenchyma by drawing a circle ROI on the right hepatic lobe, and the standard deviation (SD) of the surrounding background liver parenchyma (SD_liver_) is chosen as a more reliable estimate of image noise [1]. The median SNR of HCC was 45.74 (interquartile range, 33.65-64.9) on pre-RT DWI and was 26.32 (interquartile range, 14.45-38.26) on post-RT DWI. The median SNR of liver parenchyma was 14.48 (interquartile range, 11.33-16.87) on pre-RT DWI and was 12.44 (interquartile range, 10.24-14.90) on post-RT DWI.

References

1 Zhu Y, Wang P, Wang B et al (2025) Accelerated Multi-b-Value DWI Using Deep Learning Reconstruction: Image Quality Improvement and Microvascular Invasion Prediction in BCLC Stage A Hepatocellular Carcinoma. Acad Radiol. 10.1016/j.acra.2025.01.043

Table S1 MRI sequences and parameters

| **Sequence** | **Dual-echo T1WI** | **T2WI/FS** | **DWI** | **LAVA** |
| --- | --- | --- | --- | --- |
| **GE Signa HDx** | | | | |
| TR (ms) | 4.6 | 10,000 | 8571 | 3.9 |
| TE (ms) | 2.5/1.3 | 91.9 | 59.3 | 1.6 |
| FOV (mm) | 400×320 | 360×360 | 360×360 | 360×288 |
| Bandwidth (kHz) | 142.9 | 62.5 | 250.0 | 100.0 |
| Slice thickness /space (mm) | 5.0/0 | 6.0/1.0 | 6.0/1.0 | 4.8/0 |
| Matrix (phase×frequency) | 256×224 | 320×320 | 128×128 | 288×192 |
| **GE Discovery MR 750** | | | | |
| TR (ms) | 3.9 | 6,000 | 6316 | 3.1 |
| TE (ms) | 2.2/1.2 | 85.6 | 58.8 | 1.3 |
| FOV (mm) | 360×288 | 360×304 | 380×304 | 360×288 |
| Bandwidth (kHz) | 200.0 | 83.3 | 250.0 | 125.0 |
| Slice thickness/space (mm) | 5.0/0 | 6.5/1.0 | 6.5/1.0 | 5.0/0 |
| Matrix (phase×frequency) | 256×224 | 320×224 | 128×160 | 256×192 |

Abbreviation: T1WI, T1-weighted imaging; T2WI, T2-weighted imaging; FS, fat suppression; DWI, diffusion-weighted imaging; LAVA, liver acquisition with volume acceleration; TR, repetition time; TE, echo time; FOV, field of view.

Results

Table S2 Intraclass correlation coefficients for ADC parameters

|  | Pre-RT | Post-RT |
| --- | --- | --- |
| ADC_roi_ | 0.774 | 0.850 |
| ADC_slice_ | 0.890 | 0.865 |
| ADC_mean_ | 0.984 | 0.951 |
| Other histogram metrics | 0.772-0.997 | 0.721-0.973 |

Abbreviation: ADC, apparent diffusion coefficient; RT, radiotherapy.


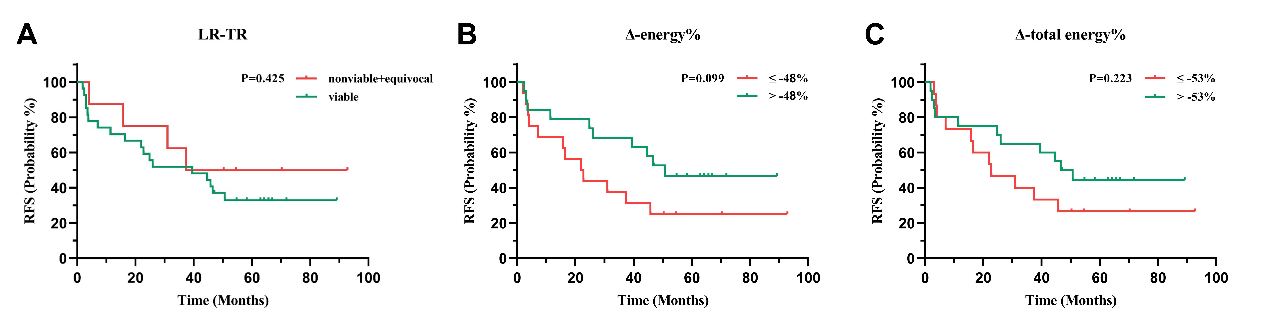


Fig. S1. Kaplan–Meier curves for RFS of 35 patients grouped by LR-TR (A), Δ-energy% (B), and Δ-total energy% (C).
